# Correction: Overlapping communities detection through weighted graph community games

**DOI:** 10.1371/journal.pone.0296580

**Published:** 2024-01-02

**Authors:** Stefano Benati, Justo Puerto, Antonio M. Rodríguez-Chía, Francisco Temprano

There are errors in the Funding section. The correct Funding statement is: The authors of this research Stefano Benati, Antonio Manuel Rodríguez-Chía, Justo Puerto and Francisco Temprano acknowledge financial support by the reasearch project PID2020-114594GB-C21 funded by the Spanish Ministerio de Ciencia e Innovación and Agencia Estatal de Investigación (MCIN/ AEI /10.13039/501100011033). The authors also acknowledge partial support from: Junta de Andalucía with grant number: P18-FR-1422, NetmeetData: Ayudas Fundación BBVA a equipos de investigación científica 2019 with reference "COMPLEX NETWORK". The author Antonio Manuel Rodríguez-Chía also acknowledges the European Regional Development Fund via projects with grant numbers: FEDER-UCA18-106895 and TED2021-130875B-I00; and the Spanish Ministerio de Ciencia e Innovación and Agencia Estatal de Investigación with grant number: PID2020-114594GB-C22. The funders had no role in study design, data collection and analysis, decision to publish, or preparation of the manuscript.
