# Supplementary material for: Overlapping communities detection through weighted graph community games
Source: PLoS One. 2023 Apr 4;18(4):e0283857. doi: 10.1371/journal.pone.0283857 (PMC10072486; doi:10.1371/journal.pone.0283857)
Supplement: S1 Appendix — Random network generator based on [16] benchmark. (PDF) [file pone.0283857.s001.pdf]

Appendix: Random networks generation

Stefano Benati[1], Justo Puerto[2], Antonio M. Rodríguez-Chía[3], Francisco Temprano[2*],

1 Dipartimento di Sociologia e Ricerca Sociale, Università di Trento, Via Verdi 26, 38122 Trento. Italy
2 IMUS. Universidad de Sevilla, Avda. Reina Mercedes, s/n, 41012 Sevilla. Spain
3 Faculty of Sciences, Universidad de Cádiz, Avda. República Saharaui, 11510 Puerto Real (Cádiz). Spain

* ftgarcia@us.es

Basically, Algorithm 1 is an extension of the generator presented in [16] for overlapping structures. Unlike this one, we introduce new parameters $1-\mu_o$, p and N_o that represent the minimum internal fraction of edges for each bridge node and each community it belongs to, the number of communities each bridge node belongs to and the number of bridge nodes, respectively. In order to ensure the minimum internal fraction of edges, we use the known configuration model, proposed in [21], over each community, and the remaining edges are distributed also by a configuration model over the node set V.

The remaining parameters of the algorithm are the same from [16] algorithm, as one see in lines $2-12$. The adjacent degrees and community sizes are generated by two different power law distribution in lines $14-38$, but we have to impose that adjacent degrees sum an even number and the community sizes sum $N+(p-1)N_o$ that is equivalent to sum one time each non-bridge node and p times each bridge node. In lines $40-66$, we assign each node to the communities it belongs to in a similar way as in [16] algorithm. Finally, as we mentioned above, in lines $67-78$ we generate the final network fulfilling the minimum fraction of internal edges by using partially different configuration models.

Algorithm 1 Overlapping LFR benchmark generator

procedure OVERLAPPING LFR BENCHMARK GENERATOR
$\gamma \leftarrow$ Exponent of degree power law distribution ▷ Initialize algorithm
$\beta \leftarrow$ Exponent of community size power law distribution
$k_{min} \leftarrow$ minimum degree
$k_{max} \leftarrow$ maximum degree
$s_{min} \leftarrow$ minimum community size
$s_{max} \leftarrow$ maximum community size
$N \leftarrow$ number of nodes
$N_o \leftarrow$ number of bridge nodes
$n_c \leftarrow$ maximum number of communities
$1 - \mu \leftarrow$ internal edge density non-bridge nodes
$1 - \mu_o \leftarrow$ internal edge density bridge nodes
$par\_degree \leftarrow False$
while $par\_degree = False$ **do**
for i in V **do**
$k_i \leftarrow power\_law(k_{min}, k_{max}, \gamma)$ ▷ Selection of degrees by a power law distribution
end for
if $\sum_{i \in V} k_i$ is par **then**
$par\_degree \leftarrow True$ ▷ Sum of adjacent degrees must be par, if not, the selection restart
end if
end while
$k \leftarrow 1$
$sum\_community\_sizes \leftarrow 0$
$n_{com} \leftarrow 0$
while $sum\_community\_sizes < N + (p-1)N_o$ **do**
if $N + (p-1)N_o - sum\_community\_sizes < s_{min}$ or $k > n_c$ **then**
$k \leftarrow 1$ ▷ If the last community takes less than the minimum size or the maximum number of communities is exceeded, the selection restart
$sum\_community\_sizes \leftarrow 0$
$n_{com} \leftarrow 0$
end if
$s_k \leftarrow power\_law(s_{min}, s_{max}, \beta)$ ▷ Selection of the size of each community by a power law distribution
if $sum\_community\_sizes + s_k > N + (p-1)N_o$ **then**
$s_k \leftarrow N + (p-1)N_o - sum\_community\_sizes$ ▷ If the limit is exceeded, the last community takes the remainder number of nodes
end if
$k \leftarrow k + 1$
$sum\_community\_sizes \leftarrow sum\_community\_sizes + s_k$
$n_{com} \leftarrow n_{com} + 1$
end while
$bridge\_nodes \leftarrow random\_subset(V, N_o)$ ▷ Random selection of N_o bridge nodes
for k in $\{1, \ldots, n_{com}\}$ **do**
$S_k \leftarrow \{\}$ ▷ Community k
end for
for i in $bridge\_nodes$ **do**
$C_i \leftarrow \{\}$ ▷ Community indexes to which i belongs
end for
$candidate\_nodes \leftarrow V$ ▷ Nodes that can be introduced into a community
while $|candidate\_nodes| > 0$ **do** ▷ The process ends when no nodes can be selected and all the communities are completed
$i \leftarrow random\_subset(candidate\_nodes, 1)$ ▷ Random selection of 1 node that can be introduced into a community
$k \leftarrow random\_subset(\{1, \ldots, n_c\}, 1)$ ▷ Random selection of a community to introduce a node
if i not in S_k **then** ▷ i must not belongs to S_k
$S_k \leftarrow S_k \cup \{i\}$ ▷ Introduce i into S_k
if i in $bridge\_nodes$ **then**
$C_i \leftarrow C_i \cup \{k\}$ ▷ k belongs to C_i
if $|C_i| = p$ **then**
$candidate\_nodes \leftarrow candidate\_nodes \setminus \{i\}$ ▷ Bridge node i can not belong to more than p communities
end if
else
$candidate\_nodes \leftarrow candidate\_nodes \setminus \{i\}$ ▷ Non-bridge node i can not belong to more than 1 community
end if
if $|S_k| > s_k$ **then** ▷ When a community S_k is exceed, we remove one random node from the community
$i' \leftarrow random\_subset(S_k, 1)$
$S_k \leftarrow S_k \setminus \{i'\}$
$candidate\_nodes \leftarrow candidate\_nodes \cup \{i'\}$ ▷ The removed node can be introduced into a new community
end if
end if
end while
$E \leftarrow \{\}$ ▷ Edges set of the network
for k in $\{1, \ldots, n_{com}\}$ **do**
for i in S_k **do**
if i in $bridge\_nodes$ **then**
$k\_int_i \leftarrow round((1 - \mu_o)k_i)$ ▷ Internal degree of bridge node i in S_k
else
$k\_int_i \leftarrow round((1 - \mu)k_i)$ ▷ Internal degree of non-bridge node i in S_k
end if
end for
$E \leftarrow E \cup random\_graph(S_k, \{k\_int_i : i \in S_k\})$ ▷ Configuration model apply to S_k and their internal degrees
end for
$E \leftarrow E \cup random\_graph(V, \{k_i - k\_int_i : i \in V\})$ ▷ Configuration model apply to V and their external degrees
return $G = (V, E)$
end procedure

References

1. Girvan M, Newman MEJ. Finding and evaluating community structure in networks. Phys Rev E. 2004;(69 (2), 026113).
2. Fortunato S, Hric D. Community detection in networks: A user guide. Physics Reports. 2016;659:1–44.
3. Palla G, Derényi I, Farkas I, Vicsek T. Uncovering the Overlapping Community Structure of Complex Networks in Nature and Society. Nature. 2005;(435 (7043)).
4. Xie J, Kelley S, Szymanski BK. Overlapping Community Detection in Networks: The State-of-the-Art and Comparative Study. Comput Surv. 2013;45(43):1–35.
5. Agarwal G, Kempe D. Modularity-maximizing graph communities via mathematical programming. The European Physical Journal B. 2008;66(3):409–418. doi:10.1140/epjb/e2008-00425-1.
6. Li Z, Zhang XS, Wang RS, Liu H, Zhang S. Discovering Link Communities in Complex Networks by an Integer Programming Model and a Genetic Algorithm. PLoS ONE. 2013;(8 (12), e83739). doi:https://doi.org/10.1371/journal.pone.0083739.
7. Bennett L, Kittas A, Liu S, Papageorgiou LG, Tsoka S. Community Structure Detection for Overlapping Modules through Mathematical Programming in Protein Interaction Networks. PLoS ONE. 2014;(9(11): e112821).
8. Costa A, Ng TS, Foo LX. Complete mixed integer linear programming formulations for modularity density based clustering. Discrete Optimization. 2017;(25):141–158.
9. Zhang S, Wang RS, Zhang X. Identification of overlapping community structure in complex networks using fuzzy c-means clustering. Physica A. 2007;(374):483–490.
10. Nepusz T, Petroczi A, Negyessy L, Bazso F. Fuzzy Communities and the Concept of Bridgeness in Complex Networks. Physical Review E. 2008;77:16–107.
11. Nicosia V, Mangioni G, Carchiolo V, Malgeri M. Extending the definition of modularity to directed graphs with overlapping communities. J Stat Mech Theory Exp. 2009;((03) (2009) P03024).
12. Chen D, Shang M, Fu Y. Detecting overlapping communities of weighted networks via a local algorithm. Physica A: Statistical Mechanics and its Applications. 2010;(389):4177–4187.
13. Chitra Devi J, Poovammal E. An Analysis of Overlapping Community Detection Algorithms in Social Networks. Procedia Computer Science. 2016;(89):349–358.
14. Benati S, Puerto J, Rodríguez-Chía AM, Temprano F. A mathematical programming approach to overlapping community detection. Physica A: Statistical Mechanics and its Applications. 2022;602:127628.
15. Jonnalagadda A, Kuppusamy L. A cooperative game framework for detecting overlapping communities in social networks. Physica A. 2018;(491):498–515.
16. Lancichinetti A, Fortunato S, Radicchi F. Benchmark graphs for testing community detection algorithms. Phys Rev E. 2008;78:046110.
17. Demange G. Intermediate preferences and stable coalition structures. Journal of Mathematical Economics. 1994;23(1):45–58.

18. Carraro C, Marchiori C. Stable Coalitions. 2002;(3258).

19. D'Aspremont C, Jacquemin A, Gabszewicz JJ, Weymark JA. On the Stability of Collusive Price Leadership. The Canadian Journal of Economics / Revue canadienne d'Economique. 1983;16(1):17–25.

20. Caparros A, Giraud-Héraud E, Hammoudi A, Tazdaït T. Coalition Stability with Heterogeneous Agents. Economics Bulletin. 2011;31(1):286–296.

21. Newman MEJ. Networks: an introduction. Oxford University Press; 2010.

22. Newman MEJ. Analysis of weighted networks. Phys Rev E. 2004;(70 (5), 056131).

23. Callan D. A combinatorial survey of identities for the double factorial. 2009;.

24. Zachary WW. An Information Flow Model for Conflict and Fission in Small Groups. Journal of Anthropological Research. 1977;(33 (4)):452–473.

25. Sundaresan SR, Fischhoff IR, Dushoff J, Rubenstein DI. Network metrics reveal diVerences in social organization between two Wssion-fusion species, Grevy's zebra and onager. Oecologia. 2007;151:140–149.

26. Read KE. Cultures of the central highlands, New Guinea. Southwestern Journal of Anthropology. 1954; p. 1–43.

27. Freeman LC, Freeman SC, Michaelson AG. On human social intelligence. Journal of Social Biological Structure. 1988;11:415–425.

28. Girvan M, Newman MEJ. Community structure in social and biological networks. Proceedings of the National Academy of Sciences. 2002;99(12):7821-7826.

29. Gleiser P, Danon L. Community Structure in Jazz. Advances in Complex Systems (ACS). 2003;06:565-573.

30. Jeong H, Tombor B, Albert R, Oltvai Z, Barabasi AL. The Large-Scale Organization of Metabolic Networks. Nature. 2000;407(6804):651-654.

31. Tandon A, Albeshri A, Thayananthan V, Alhalabi W, Radicchi F, Fortunato S. Community detection in networks using graph embeddings. Phys Rev E. 2021;103:022316.

32. Lancichinetti A, Fortunato S, Kertész J. Detecting the overlapping and hierarchical community structure in complex networks. New Journal of Physics. 2009;11(3).

33. Collins LM, Dent CW. Omega: A General Formulation of the Rand Index of Cluster Recovery Suitable for Non-disjoint Solutions. Multivariate Behavioral Research. 2005;23:231–242.
