# Supplementary material for: Overlapping communities detection through weighted graph community games
Source: PLoS One. 2023 Apr 4;18(4):e0283857. doi: 10.1371/journal.pone.0283857 (PMC10072486; doi:10.1371/journal.pone.0283857)
Supplement: Attachment — Submitted filename: Response to Reviewers.pdf [file pone.0283857.s002.pdf]

Answer to Reviewers

Dear reviewers,

following the journal requirements, we have addressed the additional requirements:

(1) We have adapted our manuscript to the PLOS ONE’s style.

(2) All codes and datasets needed to reproduce our experimentation are now shared.

(3) Funding Information and Financial Disclosure sections have been corrected and matched.

(4) Acknowledgements Section has been removed.

Following referees’ suggestions:

(1) The setup is good, but the chosen datasets are very small (less 100 nodes). I suggest to add larger datasets to test the scalability of the proposal.

We have added three real datasets with 115, 198 and 453 nodes that are commonly used in literature. The ILP model is not able to certify optimality for instances with these sizes, so they have been solved by our LSE heuristic. Moreover, in Results and discussion section we solved random instances of 500 and 1000 nodes using the same algorithm.
